# Antioxidant, Anti-Apoptotic, and Anti-Inflammatory Effects of Farrerol in a Mouse Model of Obstructive Uropathy

**DOI:** 10.3390/cimb45010024

**Published:** 2023-01-01

**Authors:** Jung-Yeon Kim, Jaechan Leem, Kwan-Kyu Park

**Affiliations:** 1 Department of Immunology, School of Medicine, Daegu Catholic University, Daegu 42472, Republic of Korea; 2 Department of Pathology, School of Medicine, Daegu Catholic University, Daegu 42472, Republic of Korea

**Keywords:** farrerol, obstructive uropathy, oxidative stress, apoptosis, inflammation

## Abstract

Obstructive uropathy is a clinical condition that can lead to chronic kidney disease. However, treatments that can prevent the progression of renal injury and fibrosis are limited. Farrerol (FA) is a natural flavone with potent antioxidant and anti-inflammatory properties. Here, we investigated the effect of FA on renal injury and fibrosis in a mouse model of unilateral ureteral obstruction (UUO). Mice underwent a sham or UUO operation and received intraperitoneal injections of FA (20 mg/kg) daily for 8 consecutive days. Histochemistry, immunohistochemistry and immunofluorescence staining, TdT-mediated dUTP nick end labeling assay, Western blotting, gene expression analysis, and biochemical tests were performed. FA attenuated renal dysfunction (*p* < 0.05) and ameliorated renal tubular injury (*p* < 0.01) and interstitial fibrosis (*p* < 0.001) in UUO mice. FA alleviated 4-hydroxynonenal expression (*p* < 0.001) and malondialdehyde levels (*p* < 0.01) by regulating pro-oxidant and antioxidant enzymes. Apoptosis in the kidneys of UUO mice was inhibited by FA (*p* < 0.001), and this action was accompanied by decreased expression of cleaved caspase-3 (*p* < 0.01). Moreover, FA alleviated pro-inflammatory cytokine production (*p* < 0.001) and macrophage infiltration (*p* < 0.01) in the kidneys of UUO mice. These results suggest that FA ameliorates renal injury and fibrosis in the UUO model by inhibiting oxidative stress, apoptosis, and inflammation.

## 1. Introduction

Obstructive uropathy is a condition in which urinary flow is partially or completely blocked [1]. This condition is caused by congenital anomalies, urolithiasis, benign prostatic hyperplasia, and tumors. While obstructive uropathy is frequently associated with acute renal failure in adults, it is known to be one of the major causes of chronic kidney disease (CKD) in children [1,2]. Current treatment for obstructive uropathy consists mainly of resolving the obstruction using surgical or instrumental approaches [1]. However, despite adequate relief of obstruction, renal injury and fibrosis can progress irreversibly [3,4]. Therefore, the development of pharmacological agents for the treatment of obstructive uropathy has great clinical significance.

The obstruction in the urinary tract can cause oxidative stress, tubular cell apoptosis, and inflammation, leading to renal injury [1,5]. Renal fibrosis is a common finding in patients with chronic obstructive uropathy. In fibrotic diseases, pro-fibrogenic cytokines such as tumor growth factor-β (TGF-β) activate myofibroblasts [6,7]. These cells are specialized fibroblasts expressing α-smooth muscle actin (α-SMA) and produce large amounts of pro-inflammatory cytokines and extracellular matrix (ECM) proteins. Accumulating evidence suggests that the fibrotic process is closely associated with oxidative stress, tubular cell apoptosis, and inflammatory responses [8,9]. 

Farrerol (FA) is a natural flavone with antitussive and expectorant effects, isolated from *Rhododendron dauricum* L. [10]. Recent studies have shown that FA exhibited antioxidant and anti-inflammatory effects in rodent models of various diseases, including collagenase-induced tendinopathy [11], myocardial ischemia/reperfusion injury [12], adjuvant-induced ankle injury [13], chemical-induced colitis [14], acetaminophen-induced hepatotoxicity [15], lipopolysaccharide-induced mastitis [16], and ovalbumin-induced allergic asthma [17]. Furthermore, FA ameliorated cisplatin-induced acute kidney injury (AKI) and CKD in mice [18,19]. However, the effect of FA on obstructive uropathy has not yet been investigated. In this study, we aimed to examine the potential effects and underlying mechanisms of FA against obstructive uropathy in the unilateral ureteral obstruction (UUO) mouse model. This model has been widely used to study obstructive uropathy and is believed to mimic human CKD [20,21]. Previous studies have shown that rodents with UUO exhibit oxidative stress, apoptosis, inflammation, and fibrosis in the obstructed kidney [22,23]. Therefore, the UUO model is very useful for testing potential therapeutic agents for obstructive uropathy.

## 2. Materials and Methods

### 2.1. Animal Experiments

**Figure 1:**
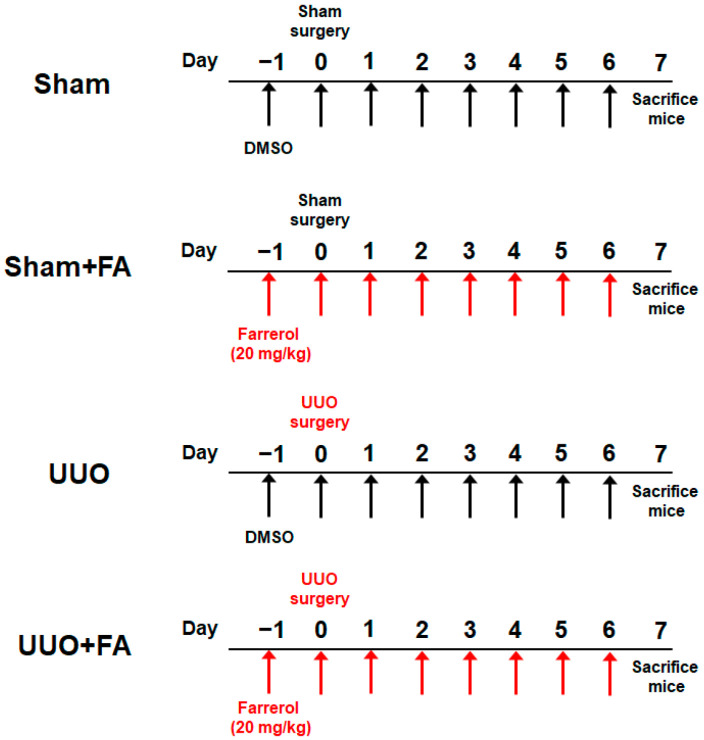
Overview of the animal experiment protocol.

Seven-week-old male C57BL/6J mice were purchased from HyoSung Science (Daegu, Korea) and maintained at 20–24 °C with a 12/12 h light/dark cycle. Drinking water and standard chow diet were provided to the mice ad libitum. After 1 week of accommodation, the mice were arbitrarily grouped into 4 groups (*n* = 8 in each group): (1) sham-operated control (Sham) group; (2) Sham + FA group; (3) UUO group; and (4) UUO + FA group. To create a ureteric obstruction, the left ureter was exposed through a flank incision and ligated with 5-0 silk sutures. Mice in the Sham and Sham + FA groups underwent a surgical procedure similar to UUO but did not undergo ureteral ligation. Mice in the Sham + FA and UUO + FA groups received intraperitoneal injections of FA (20 mg/kg) daily for 8 consecutive days, starting 1 day before the sham or UUO operation. FA was acquired from Selleckchem (Houston, TX, USA) and dissolved in DMSO. Mice in the Sham and UUO groups were injected intraperitoneally with an equal volume of DMSO. The dose of FA was chosen based on previous studies [17,18]. One week after the sham or UUO operation, all mice were anesthetized and sacrificed. The protocol is summarized in Figure 1. Animal experiments were approved by the Institutional Animal Care and Use Committee of the Daegu Catholic University Medical Center (DCIAFCR-211220-29-Y).

### 2.2. Assessment of Renal Function and Cytokine Levels

Serum levels of blood urea nitrogen (BUN) and creatinine were analyzed using an autoanalyzer (Hitachi, Osaka, Japan). Serum levels of tumor necrosis factor-α (TNF-α) and interleukin-6 (IL-6) were measured using ELISA kits (R&D Systems, Minneapolis, MN, USA) following the manufacturer’s protocols.

### 2.3. Histological Analysis, Immunohistochemical (IHC) Staining, and Immunofluorescence (IF) Staining

Kidney tissues were fixed, dehydrated, embedded in paraffin, and sectioned. The kidney sections were stained with hematoxylin and eosin (H&E), periodic acid-Schiff (PAS) and Masson’s trichrome. The tubular injury score was evaluated based on the percentage of injured tubules: 0, 0%; 1, ≤10%; 2, 11–25%; 3, 26–45%; 4, 46–75%; and 5, 76–100% [24,25]. The score of each kidney sample was analyzed in 10 random fields (×400) per sample. For IHC staining, the sections were deparaffinized and rehydrated. Following antigen retrieval, the sections were reacted with antibodies against α-SMA (Sigma-Aldrich, St. Louis, MO, USA), 4-hydroxynonenal (4-HNE; Abcam, Cambridge, MA, USA), and F4/80 (Santa Cruz Biotechnology, Santa Cruz, CA, USA). Then, the sections were incubated with secondary antibodies. Images were taken from a confocal microscope (Nikon, Tokyo, Japan). Quantification of positive staining for Masson’s trichrome, α-SMA, or 4-HNE was analyzed using IMT i-Solution DT version 9.0 (IMT i-Solution, Coquitlam, BC, Canada) in 10 random fields (×400) per sample. The number of F4/80-positive cells was counted in 10 random fields (×600) per sample. To stain the proximal tubule brush border, sections were reacted with the FITC-labeled lotus tetragonolobus lectin (LTL; Vector Laboratories, Burlingame, CA, USA). DAPI was used for nuclear staining. Quantification of positive staining for LTL was analyzed in 10 random fields (×400) per sample.

### 2.4. Western Blotting

Tissues were lysed on ice in a RIPA lysis buffer. The extracted proteins were loaded onto precast gradient polyacrylamide gels and then transferred to nitrocellulose membranes. The membranes were reacted with antibodies against α-SMA (Sigma-Aldrich, St. Louis, MO, USA), NADPH oxidase 4 (NOX4; Novus Biologicals, Littleton, CO, USA), catalase (Abcam, Cambridge, MA, USA), manganese superoxide dismutase (MnSOD; Abcam, Cambridge, MA, USA), cleaved caspase-3 (Cell Signaling Technology, Danvers, MA, USA), cleaved poly(ADP-ribose) polymerase-1 (cleaved PARP-1; Cell Signaling Technology, Danvers, MA, USA), Bax (Santa Cruz Biotechnology, Santa Cruz, CA, USA), p-IκB-α (Cell Signaling Technology, Danvers, MA, USA), IκB-α (Cell Signaling Technology, Danvers, MA, USA), p-NF-κB p65 (Cell Signaling Technology, Danvers, MA, USA), NF-κB p65 (Cell Signaling Technology, Danvers, MA, USA), and glyceraldehyde-3-phosphate dehydrogenase (GAPDH; Cell Signaling Technology, Danvers, MA, USA). Then, the membranes were probed with secondary antibodies. The bands were visualized on the iBright CL1500 Imaging System (Thermo Fisher Scientific, Waltham, MA, USA) using enhanced chemiluminescence reagents. GAPDH was used as a loading control.

### 2.5. Quantitative Real-Time Polymerase Chain Reaction (qRT-PCR)

Total RNA extraction from tissues was performed using TRIzol reagent (Sigma-Aldrich, St. Louis, MO, USA). After the RNA was reverse-transcribed into cDNA, qRT-PCR was performed using the specific primers (Table 1) in the Thermal Cycler Dice Real Time System III (TaKaRa, Tokyo, Japan). Data were calculated by the 2^−ΔΔCT^ method using GAPDH as an internal control.

### 2.6. Evaluation of Oxidative Stress and Antioxidant Enzyme Activities

Renal malondialdehyde (MDA) levels were determined using the MDA assay kit (Sigma-Aldrich, St. Louis, MO, USA). The glutathione detection kit (Enzo Life Sciences, Farmingdale, NY, USA) was used to measure the levels of reduced glutathione (GSH) and oxidized glutathione (GSSG). Activities of catalase and SOD were measured using commercial kits (Invitrogen, Carlsbad, CA, USA). Myeloperoxidase (MPO) activity was evaluated using the MPO activity assay kit (Abcam, Cambridge, MA, USA). All analyses were performed following the manufacturers’ instructions.

### 2.7. TdT-Mediated dUTP Nick End Labeling (TUNEL) Assay

TUNEL staining was conducted to detect apoptosis in tissues using a TUNEL assay kit (Roche Diagnostics, Indianapolis, IN, USA) following the manufacturer’s protocol. Positive cells were counted in 10 random fields (×600) per sample.

### 2.8. Statistical Analysis

Data were expressed as the mean ± SEM. Statistical significance was analyzed using a one-way ANOVA with Bonferroni’s multiple comparison test. *p* values < 0.05 were considered significant.

## 3. Results

### 3.1. FA Improved Renal Function and Attenuated Tubular Injury in UUO Mice

**Figure 2:**
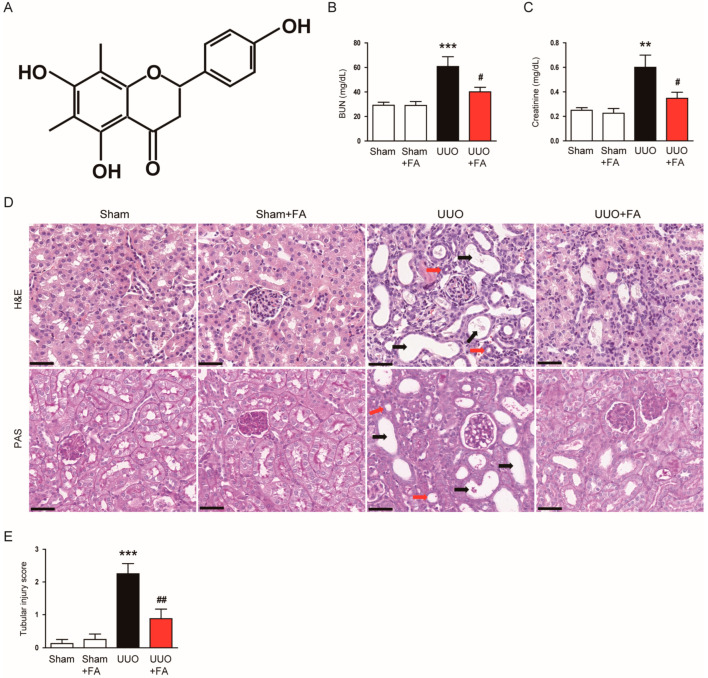
Effects of FA on renal function and histological morphology in UUO mice. (**A**) The chemical structure of FA. (**B**) BUN levels. (**C**) Serum creatinine levels. (**D**) H&E and PAS staining. Scale bar = 40 μm. Red arrows indicate tubular atrophy. Black arrows indicate tubular dilatation. (**E**) Tubular injury score. ** *p* < 0.01 and *** *p* < 0.001 vs. Sham. ^#^ *p* < 0.05 and ^##^ *p* < 0.01 vs. UUO.

To evaluate the effect of FA (Figure 2A) on renal function, serum levels of BUN and creatinine, which are important clinical indicators of renal function [26,27], were measured. The serum BUN and creatinine levels were increased after the UUO operation (BUN: Sham, 29.3 ± 2.4 mg/dL vs. UUO, 60.8 ± 8.0 mg/dL, *p* < 0.001; creatinine: Sham, 0.25 ± 0.02 mg/dL vs. UUO, 0.60 ± 0.10 mg/dL, *p* < 0.01) (Figure 2B,C). FA treatment remarkably reduced serum levels of both indicators in UUO mice (BUN: UUO, 60.8 ± 8.0 mg/dL vs. UUO + FA, 40.1 ± 3.7 mg/dL, *p* < 0.05; creatinine: UUO, 0.60 ± 0.10 mg/dL vs. UUO + FA, 0.35 ± 0.05 mg/dL, *p* < 0.05) (Figure 2B,C). Histological examination revealed that UUO mice exhibited renal tubular atrophy, tubular dilatation, and inflammatory cell infiltration (Figure 2D). When UUO mice were treated with FA, the histological abnormalities were attenuated (Figure 2D). In the UUO group, the increase in tubular injury score was significantly reduced by FA (UUO, 3.5 ± 0.3 vs. UUO + FA, 1.6 ± 0.3, *p* < 0.001) (Figure 2E).

**Figure 3:**
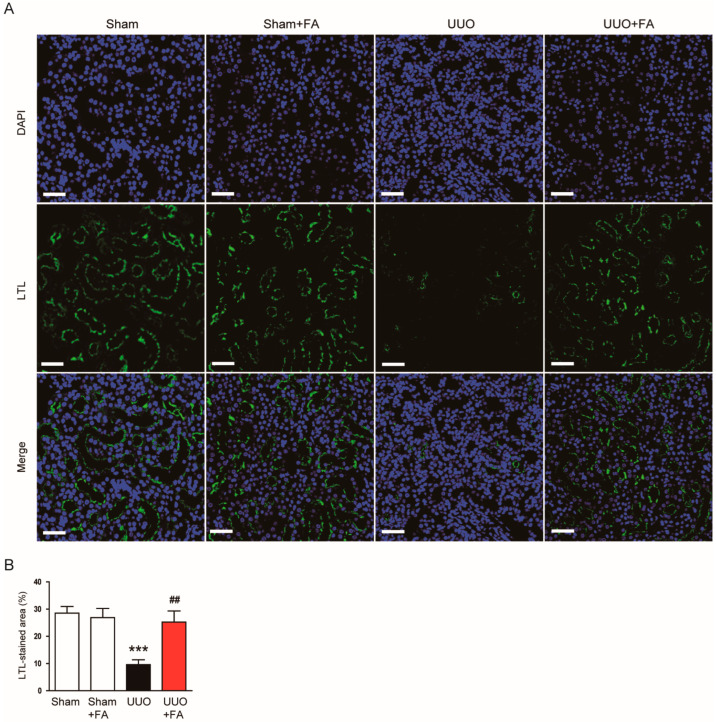
Effects of FA on loss of proximal tubule brush border in UUO mice. (**A**) IF staining for LTL. Scale bar = 50 μm. (**B**) Percentages of LTL-stained area. *** *p* < 0.001 vs. Sham. ^##^ *p* < 0.01 vs. UUO.

LTL is a specific marker for the proximal tubule brush border [28,29]. IF staining for LTL revealed that the percentage of LTL-stained area was decreased after the UUO operation (Sham, 28.5 ± 2.4% vs. UUO, 9.6 ± 1.8%, *p* < 0.001) (Figure 3A,B). When UUO mice were treated with FA, UUO-induced loss of the brush border was significantly alleviated (UUO, 9.6 ± 1.8% vs. UUO + FA, 25.3 ± 4.1%, *p* < 0.01) (Figure 3A,B).

### 3.2. FA Attenuated Renal Fibrosis in UUO Mice

**Figure 4:**
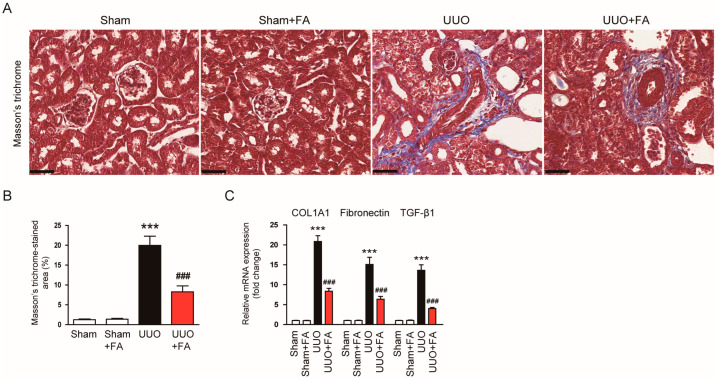
Effects of FA on renal fibrosis in UUO mice. (**A**) Masson’s trichrome staining. Scale bar = 40 μm. (**B**) Percentages of Masson’s trichrome-stained area. (**C**) Renal COL1A1, fibronectin, and TGF-β1 mRNA levels. *** *p* < 0.001 vs. Sham. ^###^ *p* < 0.001 vs. UUO.

Renal fibrosis was examined using Masson’s trichrome staining. The area of renal fibrosis was remarkably greater in UUO mice than in Sham mice (Sham, 1.3 ± 0.2% vs. UUO, 20.0 ± 2.3%, *p* < 0.001) (Figure 4A,B). FA significantly decreased the area of renal fibrosis in UUO mice (UUO, 20.0 ± 2.3% vs. UUO + FA, 8.3 ± 1.5, *p* < 0.001) (Figure 4A,B). The renal mRNA expression of collagen 1A1 (COL1A1), fibronectin, and TGF-β1 was also reduced by FA (COL1A1: UUO, 20.8 ± 1.5 vs. UUO + FA, 8.4 ± 0.7, *p* < 0.001; fibronectin: UUO, 15.1 ± 1.8 vs. UUO + FA, 6.4 ± 0.7, *p* < 0.001; TGF-β1: UUO, 13.6 ± 1.4 vs. UUO + FA, 4.0 ± 0.3, *p* < 0.001) (Figure 4C).

**Figure 5:**
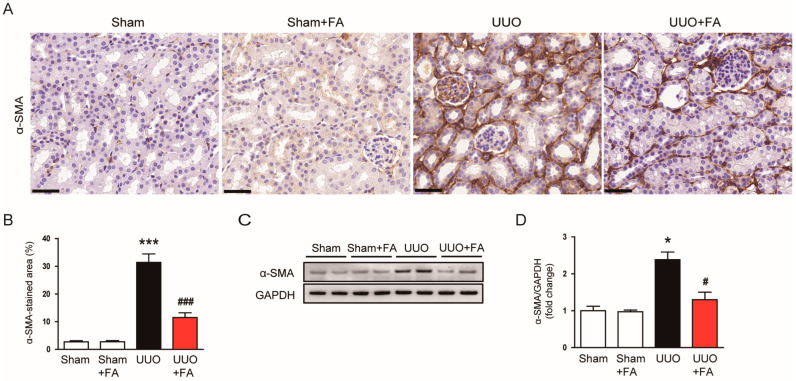
Effects of FA on myofibroblast accumulation in UUO mice. (**A**) IHC staining for α-SMA. Scale bar = 40 μm. (**B**) Percentages of α-SMA-stained area. (**C**) Western blotting of α-SMA. (**D**) Quantification of Western blots for α-SMA. * *p* < 0.05 and *** *p* < 0.001 vs. Sham. ^#^ *p* < 0.05 and ^###^ *p* < 0.001 vs. UUO.

Myofibroblasts are key effector cells in renal fibrosis, which are responsible for ECM protein production [6,7]. α-SMA is an established marker of differentiated myofibroblasts [30]. IHC staining revealed that the area of positive staining for α-SMA was largely increased after the UUO operation (Sham, 2.7 ± 0.4% vs. UUO, 31.4 ± 3.1%, *p* < 0.001) (Figure 5A,B). When UUO mice were treated with FA, the expression of α-SMA was significantly reduced (UUO, 31.4 ± 3.1% vs. UUO + FA, 11.5 ± 1.7%, *p* < 0.001) (Figure 5A,B). Western blotting also confirmed the inhibitory effect of FA on α-SMA expression (UUO, 2.4 ± 0.2 vs. UUO + FA, 1.3 ± 0.2, *p* < 0.05) (Figure 5C,D).

### 3.3. FA Suppressed Oxidative Damage in UUO Mice

**Figure 6:**
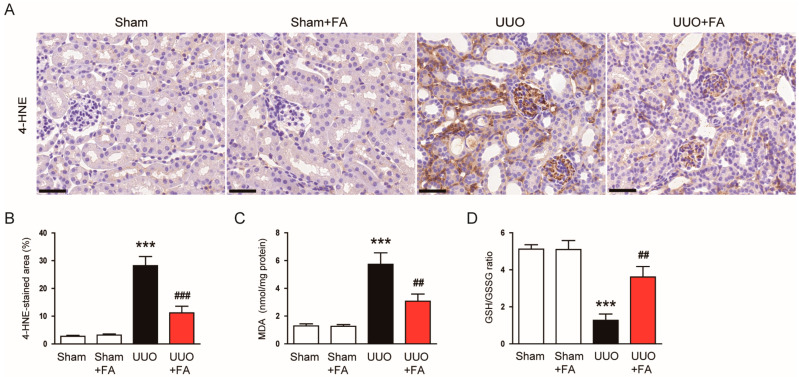
Effects of FA on oxidative stress in UUO mice. (**A**) IHC staining for 4-HNE. Scale bar = 40 μm. (**B**) Percentages of 4-HNE-stained area. (**C**) Renal MDA levels. (**D**) GSH/GSSG ratio. *** *p* < 0.001 vs. Sham. ^##^ *p* < 0.01 and ^###^ *p* < 0.001 vs. UUO.

Oxidative stress plays a crucial role in the pathogenesis of UUO [31,32]. Thus, to explore the potential mechanism of action of FA, we evaluated the effect of FA on UUO-induced oxidative stress. 4-HNE and MDA are major products of lipid peroxidation [33,34]. The area of positive staining for 4-HNE was largely increased after the UUO operation (Sham, 2.8 ± 0.3% vs. UUO, 28.2 ± 3.3%, *p* < 0.001) (Figure 6A,B). FA treatment significantly reduced 4-HNE expression in UUO mice (UUO, 28.2 ± 3.3% vs. UUO + FA, 11.2 ± 2.4%, *p* < 0.001) (Figure 6A,B). Renal MDA levels were also reduced by FA (UUO, 5.7 ± 0.8 nmol/mg protein vs. UUO + FA, 3.1 ± 0.5 nmol/mg protein, *p* < 0.01) (Figure 6C). Furthermore, FA significantly reversed a decrease in the GSH/GSSG ratio, an established indicator of oxidative stress [35] in UUO mice (UUO, 1.3 ± 0.3 vs. UUO + FA, 3.6 ± 0.6, *p* < 0.01) (Figure 6D).

**Figure 7:**
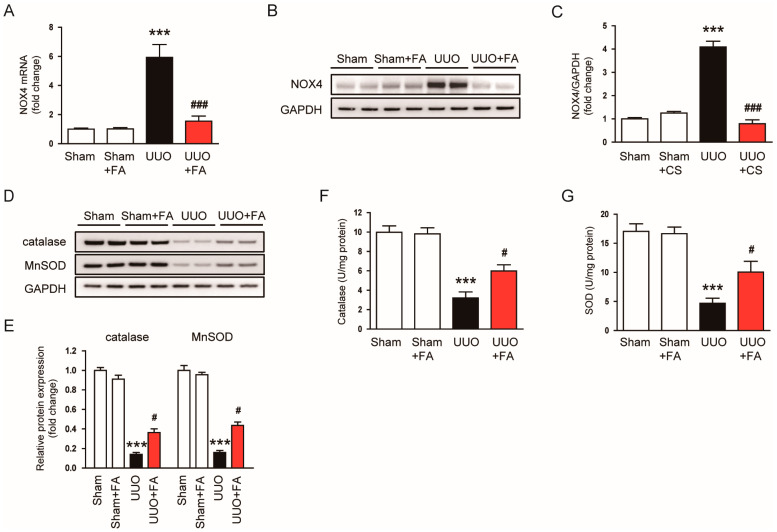
Effects of FA on pro-oxidant and antioxidant enzymes in UUO mice. (**A**) Renal NOX4 mRNA levels. (**B**) Western blotting of NOX4. (**C**) Quantification of Western blots for NOX4. (**D**) Western blotting of catalase and MnSOD. (**E**) Quantification of Western blots for catalase and MnSOD. (**F**) Catalase activities in kidney tissues. (**G**) SOD activities in kidney tissues. *** *p* < 0.001 vs. Sham. ^#^ *p* < 0.05 and ^###^ *p* < 0.001 vs. UUO.

An imbalance between pro-oxidant and antioxidant systems is known to cause oxidative stress, leading to renal injury and fibrosis [36,37,38]. Among pro-oxidant enzymes, NOX4 is known as a key player in renal fibrosis [39]. We found that the expression of NOX4 mRNA and protein was increased after the UUO operation (NOX4 mRNA: Sham, 1.0 ± 0.1 vs. UUO, 5.9 ± 0.9, *p* < 0.001; NOX4 protein: Sham, 1.0 ± 0.1 vs. UUO, 4.1 ± 0.2, *p* < 0.001) (Figure 7A–C). FA remarkably reduced renal levels of NOX4 mRNA and protein in UUO mice (NOX4 mRNA: UUO, 5.9 ± 0.9 vs. UUO + FA, 1.6 ± 0.4, *p* < 0.001; NOX4 protein: UUO, 4.1 ± 0.2 vs. UUO + FA, 0.8 ± 0.2, *p* < 0.001) (Figure 7A–C). Moreover, the protein levels of the antioxidant enzymes catalase and MnSOD were largely decreased after the UUO operation (catalase: Sham, 1.00 ± 0.03 vs. UUO, 0.14 ± 0.02, *p* < 0.001; MnSOD: Sham, 1.00 ± 0.05 vs. UUO, 0.16 ± 0.02, *p* < 0.001) (Figure 7D,E). When UUO mice were treated with FA, the expression of catalase and MnSOD was significantly restored (catalase: UUO, 0.14 ± 0.02 vs. UUO + FA, 0.37 ± 0.04, *p* < 0.05; MnSOD: UUO, 0.16 ± 0.02 vs. UUO + FA, 0.44 ± 0.04, *p* < 0.05) (Figure 7D,E). Furthermore, FA increased the enzymatic activities of these antioxidant enzymes in the kidneys of UUO mice (catalase: UUO, 3.2 ± 0.6 U/mg protein vs. UUO + FA, 6.0 ± 0.6 U/mg protein, *p* < 0.05; MnSOD: UUO, 4.7 ± 0.9 U/mg protein vs. UUO + FA, 10.1 ± 1.8 U/mg protein, *p* < 0.05) (Figure 7F,G).

### 3.4. FA Inhibited Apoptotic Cell Death in UUO Mice

**Figure 8:**
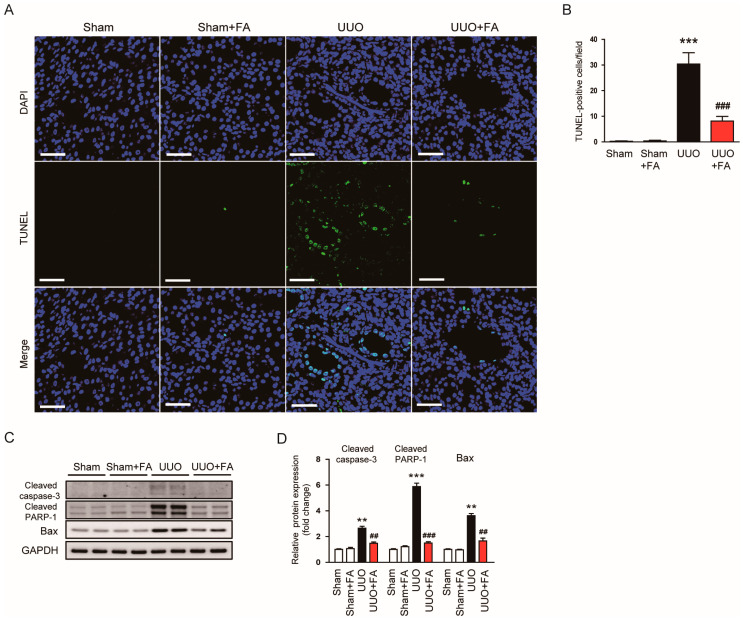
Effects of FA on apoptotic cell death in UUO mice. (**A**) TUNEL staining. Scale bar = 50 μm. (**B**) Number of TUNEL-positive cells. (**C**) Western blotting of cleaved caspase-3, cleaved PARP-1, and Bax. (**D**) Quantification of Western blots for cleaved caspase-3, cleaved PARP-1, and Bax. ** *p* < 0.01 and *** *p* < 0.001 vs. Sham. ^##^ *p* < 0.01 and ^###^ *p* < 0.001 vs. UUO.

Renal cell apoptosis also plays an important role in obstructive uropathy [40]. To detect apoptotic cells, a TUNEL assay was conducted on kidney sections. The number of TUNEL-positive cells was increased after the UUO operation (Sham, 0.3 ± 0.2 vs. UUO, 30.4 ± 4.5, *p* < 0.001) (Figure 8A,B). FA remarkably reduced the number of TUNEL-positive cells in UUO mice (UUO, 30.4 ± 4.5 vs. UUO + FA, 8.1 ± 1.8, *p* < 0.001) (Figure 8A,B). The protein expression of cleaved caspase-3, cleaved PARP-1, and Bax was also significantly decreased by FA (cleaved caspase-3: UUO, 2.7 ± 0.2 vs. UUO + FA, 1.5 ± 0.1, *p* < 0.01; cleaved PARP-1: UUO, 5.9 ± 0.3 vs. UUO + FA, 1.5 ± 0.1, *p* < 0.001; Bax: UUO, 3.6 ± 0.2 vs. UUO + FA, 1.7 ± 0.2, *p* < 0.01) (Figure 8C,D).

### 3.5. FA Suppressed Inflammatory Responses in UUO Mice

**Figure 9:**
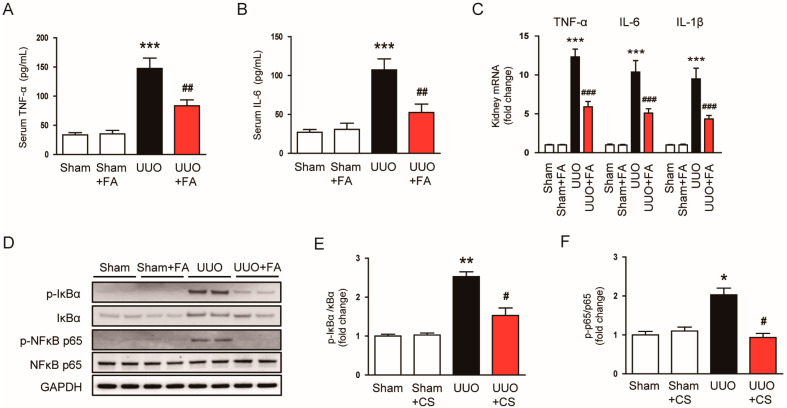
Effects of FA on cytokine production in UUO mice. (**A**,**B**) Serum TNF-α and IL-6 levels. (**C**) Renal TNF-α, IL-6, and IL-1β mRNA levels. (**D**) Western blotting of p-IκB-α and p-NFκB p65. (**E**) Quantification of Western blots for p-IκB-α. (**F**) Quantification of Western blots for p-NFκB p65. * *p* < 0.05, ** *p* < 0.01 and *** *p* < 0.001 vs. Sham. ^#^ *p* < 0.05, ^##^ *p* < 0.01 and ^###^ *p* < 0.001 vs. UUO.

Previous studies have reported an inflammatory response in the kidneys of UUO mice, which is characterized by pro-inflammatory cytokine production and immune cell infiltration [20,21]. Thus, we further explored the impacts of FA on the inflammatory responses in UUO mice. Serum levels of TNFα and IL-6 were remarkably increased after the UUO operation (TNF-α: Sham, 33.6 ± 3.8 pg/mL vs. UUO, 147.5 ± 17.8 pg/mL, *p* < 0.001; IL-6: Sham, 27.0 ± 3.7 pg/mL vs. UUO, 107.3 ± 14.1 pg/mL, *p* < 0.001) (Figure 9A,B). When UUO mice were treated with FA, serum levels of the pro-inflammatory cytokines were remarkably decreased (TNF-α: UUO, 147.5 ± 17.8 pg/mL vs. UUO + FA, 83.6 ± 10.0 pg/mL, *p* < 0.01; IL-6: UUO, 107.3 ± 14.1 pg/mL vs. UUO + FA, 52.5 ± 10.8 pg/mL, *p* < 0.01) (Figure 9A,B). FA also reduced the mRNA expression of TNFα, IL-6, and IL-1β in the kidneys of UUO mice (TNF-α: UUO, 12.3 ± 1.0 vs. UUO + FA, 5.9 ± 0.7, *p* < 0.001; IL-6: UUO, 10.4 ± 5.1 vs. UUO + FA, 5.1 ± 0.5, *p* < 0.001; IL-1β: UUO, 9.5 ± 1.4 vs. UUO + FA, 4.4 ± 0.4, *p* < 0.001) (Figure 9C). Western blot analysis was performed for p-IκBα and p-NFκB p65 to investigate the anti-inflammatory mechanism of FA. UUO mice showed increased phosphorylation of IκBα and NFκB p65 proteins compared to Sham-operated mice (p-IκBα: Sham, 1.0 ± 0.1 vs. UUO, 2.5 ± 0.1, *p* < 0.01; p-NFκB p65: Sham, 1.0 ± 0.1 vs. UUO, 2.0 ± 0.2, *p* < 0.05) (Figure 9D,E). FA significantly reduced the p-IκBα and p-NFκB p65 levels in the kidneys of UUO mice (p-IκBα: Sham, UUO, 2.5 ± 0.1 vs. UUO + FA, 1.5 ± 0.2, *p* < 0.05; p-NFκB p65: UUO, 2.0 ± 0.2 vs. UUO + FA, 0.9 ± 0.1, *p* < 0.05) (Figure 9D–F).

**Figure 10:**
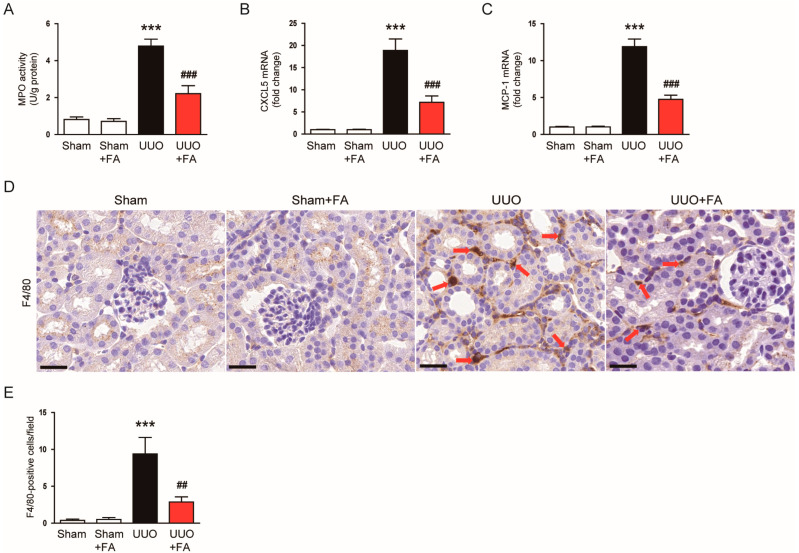
Effects of FA on macrophage infiltration in UUO mice. (**A**) Renal MPO activities. (**B**,**C**) Renal CXCL5 and MCP-1 mRNA levels. (**D**) IHC staining for F4/80. Scale bar = 60 μm. Red arrows indicate positively stained cells. (**E**) Number of F4/80-positive cells per field. *** *p* < 0.001 vs. Sham. ^##^ *p* < 0.01 and ^###^ *p* < 0.001 vs. UUO.

Macrophage infiltration is an important process in obstructive uropathy [41,42]. To examine the effect of FA on macrophage infiltration, we first measured the renal activity of MPO in each group. MPO is an enzyme secreted by activated macrophages [43]. Renal MPO activity was increased after the UUO operation (Sham, 0.8 ± 0.1 U/g protein vs. UUO, 4.8 ± 0.4 U/g protein, *p* < 0.001) (Figure 10A). When UUO mice were treated with FA, renal MPO activity was significantly inhibited (UUO, 4.8 ± 0.4 U/g protein vs. UUO + FA, 2.2 ± 0.4 U/g protein, *p* < 0.001) (Figure 10A). The expression of the chemokines C-X-C motif chemokine ligand 5 (CXCL5) and monocyte chemoattractant protein-1 (MCP-1) was also significantly reduced by FA (CXCL5: UUO, 18.9 ± 2.6 vs. UUO + FA, 7.2 ± 1.4, *p* < 0.001; MCP-1: UUO, 11.9 ± 1.0 vs. UUO + FA, 4.8 ± 0.6, *p* < 0.001) (Figure 10B,C). IF staining for F4/80, a macrophage marker [44,45], revealed that FA treatment reduced the number of F4/80-positive cells in the kidneys of UUO mice (UUO, 9.4 ± 2.2 vs. UUO + FA, 2.9 ± 0.7, *p* < 0.01) (Figure 10D,E).

## 4. Discussion

Flavones are a class of polyphenolic plant compounds that have various biological activities [46]. Among flavones, FA has long been known to have antitussive and expectorant effects [10]. However, emerging evidence from animal studies suggests that FA has a beneficial effect against several inflammatory disorders through its antioxidant and anti-inflammatory properties [13,14,15,16,17]. A recent study reported the inhibitory action of FA against cisplatin-induced AKI [18]. However, whether FA has a protective effect against obstructive uropathy has not been determined. Therefore, in this study, we investigated the effect of FA on renal injury and fibrosis in the UUO mouse model. The administration of FA attenuated renal dysfunction in UUO mice, as evidenced by reductions in serum BUN and creatinine levels. FA also ameliorated histological abnormalities such as tubular atrophy, tubular dilatation, inflammatory cell infiltration, brush border loss, and interstitial fibrosis. These results suggest that FA has a protective effect against renal injury and fibrosis in UUO mice. In this study, we also found, using IHC staining and Western blotting, that FA decreased renal α-SMA expression in UUO mice. During fibrosis, myofibroblasts express the mesenchymal marker α-SMA and produce large amounts of ECM [6,7]. These cells are activated by pro-fibrogenic cytokines. The renal mRNA expression of TGF-β1 was remarkably reduced by FA, which was accompanied by the downregulation of COL1A1 and fibronectin. Consistent with our findings, FA has been shown to attenuate cisplatin-induced renal dysfunction, tubular injury, and fibrosis in mice [19].

Accumulating evidence suggests that oxidative stress is critically implicated in the pathogenesis of obstructive uropathy [31,32]. In this study, FA treatment significantly attenuated UUO-induced oxidative stress, as evidenced by a decrease in 4-HNE-stained areas and MDA levels and an increase in the GSH/GSSG ratio. Recent studies have shown that FA attenuated oxidative injury in hydrogen peroxide-treated human retinal pigment epithelial cells [47] and vascular smooth muscle cells [48]. Cui et al. reported that FA inhibited β-amyloid-induced oxidative stress in microglial cells [49]. The antioxidant effect of FA was also observed in mouse macrophages [50]. The administration of FA alleviated oxidative stress in animal models of myocardial ischemia/reperfusion injury [12], adjuvant-induced ankle injury [13], and acetaminophen-induced hepatotoxicity [15]. Furthermore, FA attenuated oxidative stress in high glucose-exposed renal mesangial cells [51] and inhibited cisplatin-induced oxidative injury in renal tubular epithelial cells and mouse kidney [18]. Thus, the antioxidant activity of FA appears to contribute significantly to its protective effect against UUO-induced renal injury. An imbalance between pro-oxidant and antioxidant systems can cause oxidative stress, leading to renal injury and fibrosis [36,37,38]. NOX4 is the main source of reactive oxygen species in the kidney and plays an important role in renal fibrosis [39]. In this study, FA reduced renal NOX4 expression in UUO mice. A previous study showed the inhibitory effect of FA on NOX4 expression in renal tubular epithelial cells and mouse kidney [18]. Chen et al. also showed that FA exhibited antioxidant activity in high glucose-treated renal mesangial cells by downregulating NOX4 expression [51]. Thus, the antioxidant activity of FA may be mainly due to its inhibitory effect on NOX4 expression. FA also restored the reduced expression and activity of catalase and MnSOD in the kidneys of UUO mice. These antioxidant enzymes play a critical role in the prevention of UUO-induced renal injury [32]. FA has been shown to display antioxidant activity by increasing the expression and activity of antioxidant enzymes [12,52]. Taken together, our findings suggest that FA ameliorates UUO-induced oxidative injury by regulating prooxidant and antioxidant enzymes.

A recent study showed that FA attenuated cisplatin-induced CKD by inhibiting oxidative stress, inflammation, and fibrosis [19]. This effect of FA was achieved by activation of nuclear factor erythroid-2-related factor 2 (Nrf2)-mediated mitophagy. Nrf2 is a transcription factor that regulates defense systems against oxidative stress [53]. Other studies have also shown that FA can activate the Nrf2 pathway [47,48,49,50]. Therefore, further study will be required to clarify whether Nrf2 activation is also involved in the inhibitory effect of FA on UUO-induced oxidative stress.

Renal cell apoptosis also plays an important role in the pathophysiology of obstructive uropathy [40]. It has been shown that inhibition of apoptosis attenuates UUO-induced renal injury [54,55]. In this study, FA treatment remarkably inhibited UUO-induced apoptosis, as evidenced by a decreased number of TUNEL-positive cells and decreased expression of cleaved caspase-3, cleaved PARP-1, and Bax. FA is known to have anti-cancer effects, and several studies have reported that FA induces apoptosis in several types of cancer [56,57]. However, unlike cancer cells, it is reported that FA has a protective effect against apoptosis in non-cancer cells. FA inhibited apoptosis in hydrogen peroxide-treated human retinal pigment epithelial cells [47] and human vascular epithelial cells [52]. The administration of FA decreased the expression of cleaved caspase-3 and Bax in a mouse model of myocardial ischemia/reperfusion injury [12]. Thus, the anti-apoptotic effect of FA is involved in its protective effect against obstructive uropathy.

UUO mice exhibit pro-inflammatory cytokine production and immune cell infiltration [20,21]. In response to renal insults, inflammation initially acts as a defense mechanism, but long-term inflammation can promote fibrosis [58]. In this study, FA reduced serum levels of TNFα and IL-6 in UUO mice. The renal mRNA expression of TNFα, IL-6, and IL-1β was also decreased by FA. These effects of FA were accompanied by suppression of IκBα/NFκB cascade. Previous studies have reported that FA attenuated cytokine production and the NFκB pathway in mouse microglial cells [49,59]. FA reduced cytokine production in lipopolysaccharide-treated mouse macrophages [14] and human gingival fibroblasts [60] by suppressing the phosphorylation of IκBα and NFκB p65. Chen et al. also reported the suppressive action of FA on high glucose-induced cytokine production in renal mesangial cells [51]. FA inhibited inflammatory responses in IL-1β-treated human chondrocytes by blocking the NFκB signaling pathway [61]. Moreover, the administration of FA inhibited cytokine production in rodent models of several inflammatory diseases, such as adjuvant-induced ankle injury [13], chemical-induced colitis [14], and ovalbumin-induced allergic asthma [16]. In this study, we also found that FA attenuated macrophage infiltration into injured kidneys of UUO mice, as shown by reduced MPO activity and a reduced number of F4/80-positive cells. Consistently, FA reduced the renal expression of the chemokines CXCL5 and MCP-1 in UUO mice. Macrophages play a critical role in obstructive uropathy [41,42]. These cells produce many pro-inflammatory mediators and pro-fibrogenic cytokines, exacerbating renal injury and fibrosis [58]. Altogether, these results indicate that FA ameliorated UUO-induced inflammation by inhibiting cytokine production and macrophage infiltration.

In conclusion, our data demonstrated that FA treatment effectively ameliorates renal injury and fibrosis in a mouse model of obstructive uropathy by inhibiting oxidative stress, apoptosis, and inflammation. FA could be considered a potential drug candidate for the treatment of obstructive uropathy.

## Figures and Tables

**Table 1:** List of primers.

Gene	Primer Sequence (5′→3′)	Accession No.
COL1A1	F: TCCTCCAGGGATCCAACGA R: GGCAGGCGGGAGGTCTT	NM_007742
Fibronectin	F: CGAGGTGACAGAGACCACAA R: CTGGAGTCAAGCCAGACACA	NM_010233
TGF-β1	F: GCCCTGGATACCAACTATTGCTT R: AGTTGGCATGGTAGCCCTTG	NM_011577
NOX4	F: CCCAAGTTCCAAGCTCATTTCC R: TGGTGACAGGTTTGTTGCTCCT	NM_015760
TNF-α	F: CACAGAAAGCATGATCCGCGACGT R: CGGCAGAGAGGAGGTTGACTTTCT	NM_013693
IL-6	F: TAGTCCTTCCTACCCCAATTTCC R: TTGGTCCTTAGCCACTCCTTC	NM_031168
IL-1β	F: CGCAGCAGCACATCAACAAGAGC R: TGTCCTCATCCTGGAAGGTCCACG	NM_008361
CXCL5	F: TCATGAGAAGGCAATGCT R: ACATTATGCCATACTACGAAGA	NM_009141
MCP-1	F: TAAAAACCTGGATCGGAACCAA R: GCATTAGCTTCAGATTTACGGGT	NM_011333
GAPDH	F: ACTCCACTCACGGCAAATTC R: TCTCCATGGTGGTGAAGACA	NM_001289726

## Data Availability

The data supporting the findings of this study are available within the article.
